# Long-term culture of chicken tracheal organoids for the purpose of avian influenza virus research

**DOI:** 10.1186/s12985-025-02714-w

**Published:** 2025-04-15

**Authors:** Anja C. M. de Bruin, Mart M. Lamers, Bart L. Haagmans, Lonneke M. Leijten, Ron A. M. Fouchier, Mathilde Richard

**Affiliations:** 1 Department of Viroscience, Erasmus University Medical Center, Rotterdam, The Netherlands; 2 Emerging Infectious Diseases Program, Duke-NUS Medical School, Singapore, Singapore; 3 Present Address: Institute of Virology, Medical University Innsbruck, Innsbruck, Austria

**Keywords:** Organoids, Avian, Respiratory tract, HPAIV, LPAIV, Chickens, Ducks

## Abstract

There is an increasing need for reproducible long-term in vitro primary cell culture systems that are representative of the avian respiratory tract to study pathogens like highly pathogenic avian influenza viruses (HPAIVs), which threaten poultry, wildlife, and human health. Self-renewing organoid cultures allow for long-term culture, due to the presence of tissue-resident stem cells, and can approximate the in vivo cellular diversity and organization of tissues. Efforts to establish avian organoid cultures have been limited to the intestinal tract. Here, we describe the isolation and long-term culture of chicken tracheal organoids (CTOs). The CTO cultures were passaged for three to four months and cryopreserved at different stages. Mucociliary differentiation of CTOs was promoted by culture at air–liquid-interface, after which the pseudostratified epithelial cell layer of the avian trachea was recapitulated, including ciliated, goblet, and basal cells. Inoculation of CTO-derived 2D cultures with low pathogenic avian influenza viruses (LPAIVs) and HPAIVs showed that the appropriate receptors, as confirmed by virus histochemistry, and proteases to sustain multi-cycle replication of LPAIVs were expressed and that HPAIVs preferentially disseminated to the endothelium of epithelial/endothelial co-culture systems. Taken together, CTOs represent a useful tool for research on the avian respiratory tract, and their application will generate new insights into host–pathogen interactions, including HPAIV tropism.

**Supplementary Information:**

The online version contains supplementary material available at 10.1186/s12985-025-02714-w.

## Introduction

Organoids are self-renewing and self-organizing three-dimensional (3D) cultures that partially mimic the cellular diversity within and architecture of particular tissues. Their self-renewing capacity is based on the presence of stem cells, which are cultured in the presence of specific growth factors allowing to retain their stemness and therefore promoting long-term expansion. They are derived from pluripotent stem cells (i.e., embryonic stem cells or induced pluripotent stem cells) or tissue-resident multipotent stem cells, which can be of adult or fetal origin and are defined by their intrinsic organ-specific properties [1, 2]. Since the pioneering work on the establishment of intestinal organoids and their culture by Sato et al*.* [3] amongst others, the range of tissues and species from which organoid cultures have been established has increased exponentially. This now includes organoids generated from a wide range of domesticated animals for the purpose of veterinary research [4].

The global demand for poultry products has skyrocketed to the point that the estimated biomass of poultry is about threefold that of wild birds [5]. Such large poultry populations are inherently plagued with a large number of infectious diseases, caused by various viral, bacterial, and parasitic pathogens [6]. Currently, certain avian influenza viruses (AIVs) pose a threat to the poultry industry, wild bird populations, and ultimately humans through their zoonotic potential [7]. Detailed studies on host–pathogen interactions in birds are limited due to the lack of representative and reproducible in vitro cell culture systems. Efforts to establish avian organoid cultures have largely focused on the intestinal tract, yielding enteroid and enterosphere cultures of varying number of cell types, differentiation status, and longevity [8–13]. However, the tropism of pathogens such as AIV includes the respiratory tract of birds and therefore protocols for the long-term culture of cells from the avian airways are desired. Thus far, Esnault et al*.* and Imai-Matsushima et al*.* have setup long-term chicken pneumocyte type 2-like cell cultures without or with the addition of feeder cells respectively [14, 15]. Furthermore, Shen et al*.* have cultured chicken tracheal epithelial cells for three to five passages [16].

None of the abovementioned methods allow for the long-term expansion of avian respiratory epithelial cells that also recapitulate the pseudostratified tracheal epithelial cell layer. Human airway organoid (AO) cultures can be passaged for over one year, while retaining comparable ciliated, basal, club, and secretory cell frequencies [17]. Airway organoids can also be used as a source of basal cells for further mucociliary differentiation under air–liquid-interface (ALI) conditions [18–20]. Here, we established chicken tracheal organoid (CTO) cultures that were maintained for at least 15 passages and were differentiated into pseudostratified layers containing the three major cell types within the avian tracheal epithelium: ciliated, goblet, and basal cells. Furthermore, we provide proof of concept that CTO-derived differentiated 2D cultures are a useful tool for research on avian pathogens, such as low and highly pathogenic avian influenza viruses (LPAIVs; HPAIVs).

## Material and methods

### Medium formulations

The AO medium formulation is based on Sachs et al*.* [17] and consists of basal medium (Advanced DMEM/F12 (Thermofisher Scientific), 1 × GlutaMAX (Thermofisher Scientific), 10 mM HEPES (Capricorn), 100 U/mL penicillin and 100 µg/mL streptomycin (Capricorn)) supplemented with 50 µg/mL primocin (Invivogen), 25 ng/mL recombinant FGF-7 (Peprotech), 100 ng/mL recombinant FGF-10 (Peprotech), 100 ng/mL recombinant Noggin (Peprotech), 500 nM A83-01 (Tocris Bioscience), 5 µM Y-27632 (Medchem Express), 10 µM SB202190 (Merck), 1 × B27 supplement (Thermofisher Scientific), 1.25 mM N-acetylcysteine (Merck), 5 mM nicotinamide (Merck), and a 1:5 dilution of R-Spondin 1 conditioned medium. Pneumacult-ALI (StemCell) was prepared according to manufacturer’s instructions with the addition of 50 µg/mL primocin.

### Production of R-Spondin 1 conditioned medium

Cultrex HA-R-Spondin1-Fc cells (Bio-Techne), which secrete murine R-Spondin 1, were cultured for 2 passages in basal medium with the addition of 10% fetal calf serum (FCS; Sigma) and 300 µg/mL Zeocin selection reagent (Gibco). Following the 3rd passage, the cells were cultured without selection reagent. After 1 week, the medium was cleared by centrifugation (1000xg for 10 min), filtered (0.2 µm filter; Nalgene), aliquoted, and stored at − 80 °C.

### Avian tracheal organoid isolation and maintenance

A detailed step-by-step protocol for the isolation and maintenance of avian tracheal organoids is provided in Additional File 1. Briefly, tracheas were harvested from 18-day-old chicken (*Gallus gallus domesticus*) or 21- to 23-day-old domestic duck (*Anas platyrhynchos domesticus*) embryos. Basal membranes from pooled tracheas were digested with dispase (Corning) and further dissociated using mechanical force. The sample was passed through a 100 μm sieve. Single cell contaminants were removed by repeated centrifugation at 100xg followed by aspiration of the supernatant. The pellet was resuspended in Cultrex Reduced Growth Factor basement membrane extract (BME) Type 2 Select (R&D systems). BME domes were overlaid with AO medium. Tracheal organoids were cultured at 39 °C and 5% CO_2_. Tracheal organoids were passaged following BME digestion by dispase and mechanical force. Tracheal organoids were cryopreserved in Cryostor CS10 freezing medium (StemCell).

### Chicken tracheal organoids differentiation at ALI

A detailed protocol of Transwell (TW) seeding and differentiation of chicken tracheal organoids is provided in Additional File 1. Briefly, CTOs were dissociated with TrypLE Express (ThermoFisher Scientific) and seeded in AO medium on fibronectin-coated (5 µg/cm^2^) polycarbonate TWs (Corning Costar; 24-well; 6.5 mm diameter; 0.4 µm pore). On day 2 post seeding, the apical and basolateral medium was replaced with 1:1 PneumaCult-ALI:AO medium. When the cell layer reached confluency, the basolateral medium was replaced with PneumaCult-ALI and the cells were cultured at ALI. Medium was replaced every 2 days, and the cells were washed with DPBS with Ca^2+^and Mg^2+^ (ThermoFisher Scientific) every 6 days. CTO-derived 2D cultures were cultivated at 39 °C and 5% CO_2_.

### Organoid size quantification

Cultured organoids in BME domes were imaged on a Primovert inverted microscope using ZEN software (ZEISS) and surface areas of the organoid sections were determined manually with FIJI software (NIH; ImageJ version 2.1.0). All organoids in the field of view that contained a lumen and did not extend outside of the borders of the image were included in the analysis.

### Dextran permeability assay

To the apical compartment of a TW, containing 200 µL medium, 20 µL of a 100 µg/mL stock of 4 kDa dextran-FITC (Sigma Aldrich) was added. As positive control, one fibronectin-coated TW without cells was included. After 2 h of incubation at 39 °C and 5% CO_2_, 50 µL sample was collected from the basolateral compartment, containing 700 µL medium, and added to 200 µL PBS in a clear bottom microplate. Fluorescence (excitation at 485 nm and emission at 535 nm) was measured on an Infinite F200 microplate reader (TECAN). A dextran-FITC standard curve was utilized to calculate the FITC-dextran concentration in the basolateral medium, from which percentage of dextran leakage was calculated relative to the leakage through the positive control TW.

### TEER measurements

TWs were equilibrated at RT for 20 min with 200 µL medium in the apical and 500 µL medium in the basolateral compartment. TEER was measured with the EVOM2 using STX3 electrodes (World Precision Instruments), which were decontaminated with 70% ethanol and equilibrated with PBS and medium before use. Three replicate measurements were collected per TW, averaged, and the baseline resistance value from one TW without cells was subtracted. The TEER value was normalized for TW surface area, i.e., 0.33 cm^2^, and expressed as ohm*cm^2^.

### Immunofluorescence

Organoids were fixed directly in BME domes for 20 min at RT with 4% paraformaldehyde (PFA), washed thrice with PBS, resuspended in heated 2% agarose (w/v) dissolved in PBS, and processed as formalin-fixed paraffin-embedded (FFPE) samples. CTO-derived 2D cultures on TWs were fixed for 20 min at RT with 4% PFA, washed thrice with PBS, and processed as FFPE or non-FFPE samples. For processing as FFPE samples, the TWs were cut in half and placed in a drop of heated 2% agarose (w/v) in PBS and processed as FFPE. Subsequently, thin (3 µm) sections were prepared for H&E, PAS, and the immunofluorescent (IF) detection of cellular markers. Adult chicken FFPE trachea was included as positive control. For IF analysis, FFPE sections were rehydrated, and antigen was retrieved by boiling thrice for 5 min in 10 mM of citrate buffer (pH = 6). FFPE sections were washed twice with PBS with 0.5% TWEEN-20 (Sigma Aldrich) and blocked for 30 min with 10% normal goat serum (NGS; ThermoFisher Scientific) in PBS. For non-FFPE IF analysis of fixed TWs, TWs were blocked and permeabilized for 1 h at RT in PBS with 0.01% NGS, 1% bovine serum albumin (BSA), 0.1% Triton X-100 (Merck), and 0.05% TWEEN, followed by 2 washes in PBS. For IF stainings of both FFPE and non-FFPE TWs, primary and secondary antibodies were diluted in PBS with 1% BSA and incubations were performed as listed in Table 1. FFPE sections were washed thrice with PBS 0.5% TWEEN or PBS after incubation with unconjugated and conjugated antibodies respectively. Non-FFPE TWs were washed thrice with PBS after each staining. For both FFPE and non-FFPE samples, nuclei were counterstained with Hoechst 33342 (ThermoFisher Scientific) for 10 min at RT and samples were mounted under glass coverslips with Prolong Diamond Antifade Mountant (ThermoFisher Scientific). Samples were imaged on an LSM700 confocal microscope using ZEN software (Zeiss).

**Table 1:** Characteristics of antibodies used for immunofluorescence stainings

Target	Antibody	Host/Isotype	Dilution (final concentration)	Incubation
Ciliated cells	Acetylated-α-tubulin (AF647), clone 6-11B-1, Cat. No. SC-23950 (Santa Cruz)	Mouse IgG2β	1:100 (2 µg/mL)	1 h at RT
Goblet cells	Gastric muc5AC, clone 45M1, Cat. No. M5293 (Sigma Aldrich)	Mouse IgG1	1:780 (10 µg/mL)	Overnight at 4 °C
Tight junctions	ZO-1 (AF555), clone 1A12, Cat. No. MA3-39,100 (ThermoFisher Scientific)	Mouse IgG1	1:100 (10 µg/mL)	1 h at RT
Tight junctions	Occludin, Cat. No. 71–1500 (ThermoFisher Scientific)	Rabbit polyclonal	1:100 (2.5 µg/mL)	Overnight at 4 °C
Tight junctions	Claudin 5, Cat. No 34–1600 (ThermoFisher Scientific)	Rabbit polyclonal	1:100 (2.5 µg/mL)	Overnight at 4 °C
Basal cells	P63, clone 4A4, Cat. No. ab735 (Abcam)	Mouse IgG2α	1:50 (0.5 µg/mL)	Overnight at 4 °C
Mouse IgG	Goat anti-Mouse IgG (H + L) Cross-Adsorbed (AF488), Cat. No. A-11001 (ThermoFisher Scientific)	Goat polyclonal	1:400 (5 µg/mL)	1 h at RT
Rabbit IgG	Goat anti-Rabbit IgG (H + L) Highly Cross-Adsorbed (AF488), Cat. No. A11034 (ThermoFisher Scientific)	Goat polyclonal	1:400 (5 µg/mL)	1 h at RT

### Virus histochemistry

The pattern of virus attachment to CTO-derived epithelium was determined by virus histochemistry [21]. Human H3N2 influenza virus (recombinant virus produced by reverse genetics using the hemagglutinin (HA) gene of A/Netherlands/213/2003 and seven remaining genes of A/Puerto-Rico/8/1934, and passaged three times in MDCK cells) and avian H5N1 influenza virus (wild-type isolate A/turkey/Turkey/1/2005, passaged at least two times in embryonated eggs and one time in MDCK) were formalin inactivated and labelled with fluorescin isothiocyanate (FITC). FFPE sections of TWs (processed as described above under the “Immunofluorescence” section) and of tissues (adult chicken trachea, ferret nasal turbinates and cat lung) were deparaffinised in xylene and hydrated using graded alcohols. Ferret nasal turbinates and cat lung were included as controls for binding of human and avian viruses respectively [22, 23]. Adult chicken trachea was included for direct comparison with the CTO-derived epithelium. Endogenous peroxidases were blocked with 3% H_2_O_2_ diluted in PBS for 10 min at room temperature. After two washes with PBS, a blocking step with a Tris-NaCl-blocking buffer (TNB buffer, 0.5% of blocking reagent (Perkin Elmer) in 0.1 M Tris HCl, 0.15 M NaCl, pH = 7.5) for 30 min at room temperature was performed. Fifty hemagglutination units of formalin inactivated and FITC-labelled influenza viruses were incubated on the slides overnight at 4 °C in TNB buffer. After two washes with PBS-0.05% Tween, FITC was detected with a peroxidase-labelled rabbit–anti-FITC (DAKO, P5100) diluted 1/100 in TNB buffer for 1 h at room temperature. After two washes with PBS-0.05% Tween, the signal was amplified using a tyramide amplification system (Perkin-Elmer) according to the manufacturer’s instructions. After two washes with PBS-0.05% Tween, slides were incubated with Horseradish Peroxidase (HRP) coupled anti-streptavidin antibody (DAKO, D0397) diluted 1/300 in TNB buffer for 30 min at room temperature. After two washes with PBS, HRP was revealed using 3-Amino-9-Ethylcarbazole (AEC, Sigma-Aldrich) in N,N-dimethylformamide (Honeywell Fluka) diluted in a final concentration of 0.0475 M of sodium acetate (NaAc, pH = 5) with 0.05% of H_2_O_2_ for 10 min at room temperature, resulting in a bright red precipitate. A counterstain was performed with hematoxylin and the slides were embedded using Kaiser’s glycerol gelatin (Merck). Pictures were taken using an Axio Imager A2 microscope (Zeiss), a Digital Microscopy Camera Axiocam 305 (Zeiss) and acquisition with the Zen 2.3 SP1 Blue (Zeiss). The white balance of the pictures was adjusted using Adobe Photoshop.

### Co-culture of CTO-derived 2D cultures with chicken aortic endothelial cells

Chicken aortic endothelial cells (chAEC) were prepared as described previously [24, 25] and maintained in Microvascular Endothelial Cell Growth Medium-2 (EGM-2MV; LONZA) on plates coated with 0.2% gelatin (Sigma-Aldrich). For establishment of the epithelial/endothelial co-culture, CTOs were first seeded and differentiated on TWs. On day 12 post-seeding, the basolateral side of the TWs was coated by adding 500 µL 0.2% gelatin in DPBS with Ca^2+^and Mg^2+^ to the basolateral compartment and incubating for 10 min at RT. The TWs were inverted and 75,000 chAEC (passage 15) in 50 µL EGM-2MV were allowed to attach to the inverted TWs for 2 h at 39 °C and 5% CO_2_. The TWs were placed back into plates, washed once by adding 500 µL DPBS with Ca^2+^and Mg^2+^ to the basolateral compartment, and cultured at ALI with 500 µL 1:1 PC-ALI:EGM-2MV medium in the basolateral compartment. Virus inoculation of co-cultures was performed 2 days post-seeding of the chAEC.

### Inoculation of CTO-derived 2D mono- and co-cultures with avian influenza viruses

HPAIV A/chicken/Netherlands/1/03 (H7N7) and its LPAIV (HPAIV ΔMBCS) variant, with cleavage site reverted to the monobasic KGR*G, were generated by reverse genetics as previously described [26] and propagated for 2 passages in embryonated chicken eggs. Full genome sequence of the virus stocks was confirmed by Sanger sequencing. Infectious virus titers were determined by end-point titration in MDCK cells and expressed as median tissue culture infectious dose (TCID_50_/mL), as previously described [27]. Prior to TW inoculation, the apical compartment was washed once with infection medium, 1:1 PC-ALI:EGM-2MV without FCS, and 500 µL infection medium was added to the basolateral compartment. The TWs (14 dps) were inoculated apically at an MOI of 0.0001, based on the presence of 100,000 cells, by incubating with virus dilutions in 100 µL of infection medium at 39 °C and 5% CO_2_ for 30 min. CTO-derived 2D cultures were then washed twice with DPBS with Ca^2+^ and Mg^2+^. For detection of infectious virus, apical washes, harvested upon 10 min incubation with 200 µL of infection medium at RT, and basolateral samples, consisting of 100 µL of basolateral medium that was replenished afterwards, were collected. Samples were stored at − 80 °C until infectious virus titer determination. At 48 hpi, both sides of the TWs were washed once with DPBS with Ca^2+^and Mg^2+^ and fixed in 10% neutral buffered formalin. All infection experiments were performed under biosafety level 3 + conditions at the Erasmus Medical Center, Rotterdam, The Netherlands.

### Immunohistochemistry detecting the influenza virus nucleoprotein

The TWs from infection experiments were fixed in 10% neutral buffered formalin for 3 days, processed as FFPE as described above, and thin (3 µm) sections were prepared for H&E and the immunohistochemical (IHC) detection of the influenza virus nucleoprotein (NP). Sections were rehydrated and antigen retrieval was performed by treatment with 0.1% protease XIV from Streptomyces griseus (Sigma-Aldrich) in PBS for 10 min at 37 °C, followed by treatment with 3% H_2_O_2_ in PBS for 10 min at RT to block endogenous peroxidase. Sections were stained for 1 h at RT with 5 µg/mL anti-NP antibody (H16-L10-4R5 (ATCC HB-65)) or mouse IgG2α isotype control (MAB003; R&D Systems) diluted in PBS with 0.1% BSA, washed thrice with PBS 0.05% TWEEN, stained for 1 h at RT with 10 µg/mL goat-anti-mouse-IgG2α HRP (Star133P; Bio-Rad) in PBS with 0.1% BSA, and washed twice with PBS. Signal revelation was achieved by incubation for 10 min at RT with AEC (Sigma-Aldrich) in N,N-dimethylformamide (Honeywell Fluka) diluted in a final concentration of 0.0475 M of NaAc (pH = 5) with 0.05% of H_2_O_2_. Sections were counterstained with hematoxylin and mounted with Kaiser’s glycerol gelatin (VWR). Pictures were taken on the Microscope Axio Imager.A2 using ZEN software (Zeiss) and white balance was adjusted with Adobe Photoshop 2021.

## Results

### Method to produce tracheal organoids from chicken and duck embryos

**Fig. 1:**
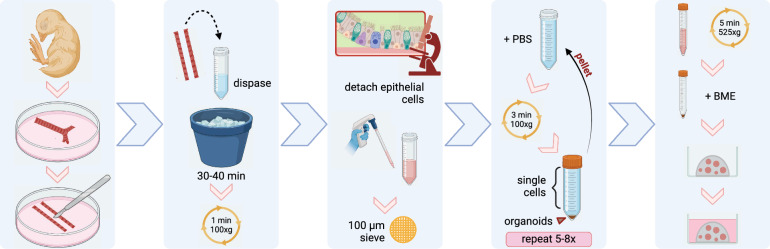
A protocol to produce embryonic avian tracheal organoid cultures. Briefly, tracheas from 18-day-old chicken or 21- to 23-day old duck embryos were dissected, cut longitudinally, pooled, and treated with dispase to dissociate the epithelial cell lining. Epithelial cells were further dissociated by repeated pipetting. The sample was sieved, and epithelial cell sheets were separated from single cell contaminants by repeated centrifugation at low speed in phosphate buffered saline (PBS). The purified epithelial cells in basal medium were pelleted and resuspended in basement membrane extract (BME). Domes of 30 µL were assembled on 48-well plates and 200 µL airway organoid medium was added after solidification. Yellow circle icons indicate centrifugation steps. Created with BioRender.com

We set out to isolate tissue-resident stem cells from the respiratory tract, i.e., basal cells, from chickens (*Gallus gallus domesticus*) and domestic ducks (*Anas platyrhynchos domesticus*), which were used as representative model species for poultry and wild bird populations respectively. Tracheas were collected from 18 day-old chicken or 21- to 23-day-old domestic duck embryos, corresponding to gestational stages 43 to 44 of the avian embryonic development [28]. The use of embryonic tissues subverts the need for sacrificing adult animals. The protocol for isolating epithelial cell sheets from the tracheal lumen was based on multiple epithelial cell isolation protocols (Fig. 1) [16, 17, 19] and a step-by-step protocol for the isolation and maintenance of avian respiratory organoids is provided in Additional File 1. Briefly, the basal membrane from pooled tracheas was digested with dispase and the epithelial cell lining was further dissociated using mechanical force. Leftover pieces of trachea were sieved out and single cell contaminants, such as erythrocytes, fibroblasts, and immune cells, were removed by repeated centrifugation at low speed. The resulting pellet, consisting of epithelial cell sheets, was resuspended in basement membrane extract (BME). The BME domes were cultured submerged in AO medium, containing growth factors to activate or block specific signaling pathways important for promoting stemness of the airway epithelium such as R-spondin 1, Noggin, and Fibroblast Growth Factors 7 and 10 [17]. Organoids were cultured at 39 °C, as avian species have higher body temperatures compared to mammals. Organoids would form cystic spheres within approximately 6 h.

### Proliferation and characterization of chicken and duck tracheal organoids

**Fig. 2:**
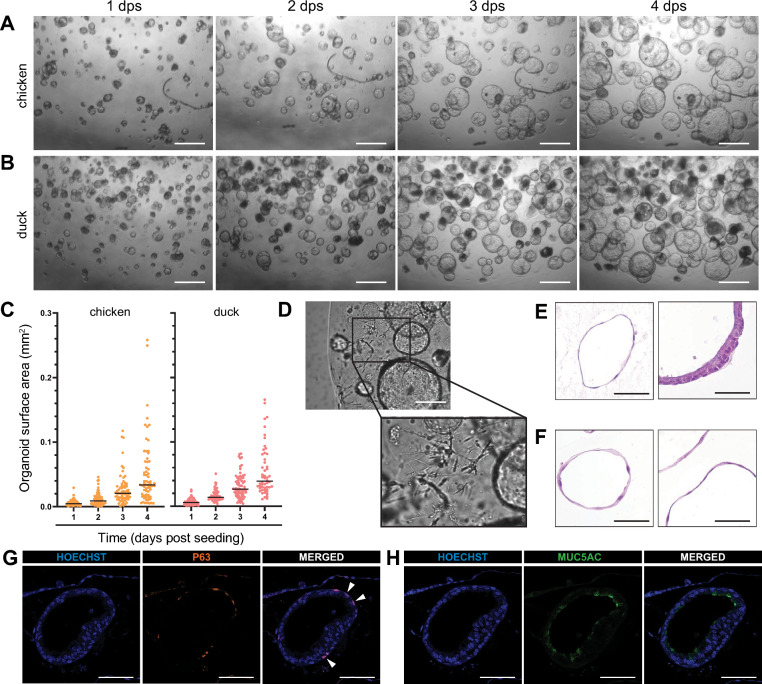
Characterization of avian tracheal organoids. **A**, **B** Growth of organoids of chicken (**A**) and duck (**B**) origin directly after isolation from 1 to 4 days post-seeding (dps). The scale bar indicates 500 µm. **C** Quantification of organoid section surface areas in mm^2^ from panels (**A**, **B**) with the median indicated by the horizontal bar. **D** Example of fibroblast contaminated area in passage 1 CTOs. The scale bar represents 200 µm. The insert shows a digitally magnified field. **E**, **F** Hematoxylin and eosin staining of CTOs showing thin- and thick-walled morphologies at passage 5 (**E**) and thin-walled morphologies at passage 15 (**F**). The scale bar represents 50 µm. **G**, **H** Immunofluorescent stainings of passage 5 thick-walled CTOs. Basal cells express p63, in orange (**G**), also indicated by white arrows. Goblet cells, positive for muc5AC, are indicated in green (**H**). Hoechst, in blue (**G**, **H**), stains nuclei. The scale bar represents 50 µm

Chicken and duck tracheal organoids (DTOs) were cultured in BME domes that were submerged in AO medium. The AO medium was refreshed every three days and cultures were passaged by mechanical disruption through pipetting every week. Epithelial cell proliferation was observed, as shown by the increase in organoid surface area (Fig. 2A, B, C). Contaminating fibroblasts were visible within the domes in passage one up to three (Fig. 2D). The detection of fibroblasts in organoid cultures decreased with each passage, due to the passaging protocol including a single cell depletion step. Organoid heterogeneity was evident in the first few passages, showing organoids of different sizes, proliferation speeds, lumen morphology (clear or dense), and wall thickness (Fig. 2A, B, C, E). After five to six passages, most organoids harbored a thin-walled morphology (Fig. 2F). CTOs, originating from more than 10 isolations, were successfully passaged for three months, whereas DTOs, originating from more than six successful isolation attempts, lost their proliferative capacity after approximately one month of passaging. Cryopreservation was successful for both species at various passage numbers, with CTOs frozen below passage five virtually always resulting in proliferating cultures. Due to the suboptimal conditions for the long-term culture of DTOs, we focused our efforts regarding characterization, differentiation, and pathogen susceptibility on CTOs.

The cellular organization of the CTOs was characterized at passage five. Immunostainings for p63, a basal cell marker, muc5AC, a goblet cell marker, and acetylated-α-tubulin, a ciliated cell marker, were performed. Basal cells were detected in the outer layer of the organoid, facing the BME (Fig. 2G). Conversely, mucus-secreting cells were detected on the inner apical rim of the organoids (Fig. 2H). However, no ciliated cells were detected, indicating the suboptimal mucociliary differentiation within the 3D culture system.

### The differentiation of CTOs at ALI

**Fig. 3:**
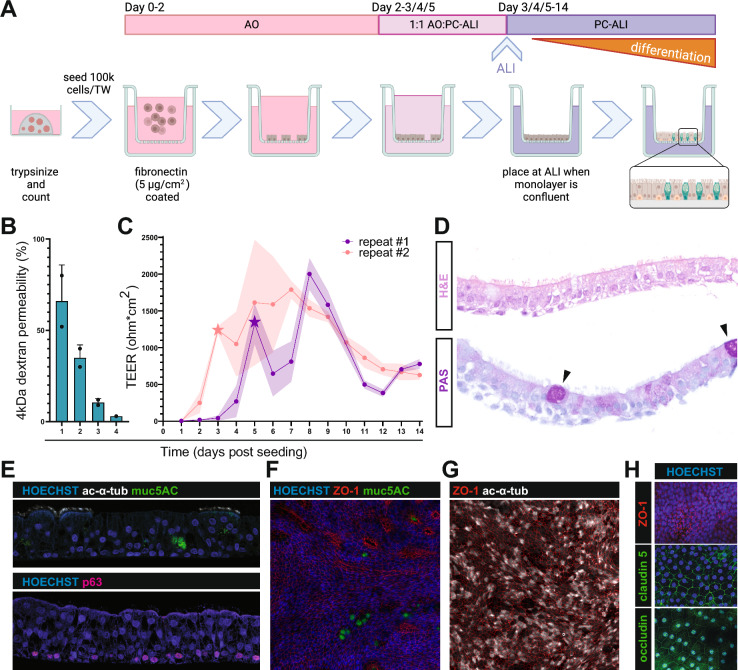
Differentiation of CTOs at ALI and their characterization. **A** Method for the seeding and differentiation of CTOs. Briefly, 100,000 cells from CTOs (passage 3–10) were seeded on a fibronectin coated Transwell (TW). The cells were cultured submerged in airway organoid (AO) medium for 2 days. The medium was then replaced by 1:1 AO and Pneumacult-air–liquid-interface (PC-ALI) medium. When confluent, the cells were brought to ALI and the medium replaced by PC-ALI medium. The medium was refreshed every 2 days until day 14, time during which the cells differentiated. Created with BioRender.com. **B** Permeability of the cell layer during the first 4 days post-seeding, indicated as leakage of 4 kDa dextran through the epithelial cell layer on TW membranes expressed as percentage of maximum leakage through an empty TW. Bars represent the mean and error bars the SD of two technical replicates performed on the same day. **C** Transepithelial electrical resistance (TEER) measurements of two independent seeding experiments. Points represent the mean and the shading the SD of six (repeat #1) and three (repeat #2) individual TWs. The star icon indicates the moment when cells were confluent and placed at ALI. **D** Hematoxylin and eosin (H&E) and Periodic Acid Schiff (PAS), indicating polysaccharide-rich mucus-containing areas, stainings of CTO-derived 2D cultures on day 14 post-seeding. The arrows mark PAS-positive goblet cells. (E–H) Immunofluorescent stainings of CTO-derived 2D cultures on day 14 post-seeding. **E** Cross section of CTO-derived 2D cultures stained for ciliated cell-marker acetylated-α-tubulin (ac-α-tub; white), goblet cell-marker muc5AC (green), basal cell-marker p63 (pink), and nuclear marker Hoechst (blue). **F**–**H** Maximum intensity projections of Z-stacks of CTO-derived 2D cultures stained with Hoechst (**F**–**H**), muc5AC (**F**), ac-α-tub (**G**), and tight junction-markers zonula occludens-1 (ZO-1; red; in (**F**–**H**)), claudin 5 (green; in (**H**)), and occludin (green; in (**H**))

To further stimulate mucociliary differentiation, CTOs were seeded on Transwell (TW) membranes and cultured at ALI (Fig. 3A). A step-by-step protocol for CTO differentiation at ALI is provided in Additional File 1. Cellular adhesion was compared between TWs coated with rat tail collagen type I, a common practice for mammalian respiratory cultures, and fibronectin by visual inspection of the monolayer. The latter significantly enhanced the seeding efficiency, which was reported before by Shen et al*.* [16]. The 4 kDa dextran permeability assay confirmed the establishment of an intact epithelial cell layer within a few days after seeding (Fig. 3B). The transepithelial electrical resistance (TEER) was monitored over the course of two weeks to assess barrier integrity of the epithelial cell layer [29]. The TEER initially increased, starting at the moment of confluency, and remained high for approximately one week before stabilizing around a value of 700 Ω*cm^2^ (Fig. 3C).

Cellular differentiation, visually assessed by the advent of ciliary beating, was observed when CTOs were cultured in Pneumacult-air–liquid-interface medium (PC-ALI) exposed to air, and usually started around seven days post-seeding (dps). The CTO differentiation efficiency was high up to passage 10, after which differentiation was not reproducible and never complete, as cilia would only appear in parts of the TW. The morphology and cellular composition of the differentiated cell layers was assessed at 14 dps, which showed a pseudostratified epithelium consisting of mostly ciliated cells with occasional goblet cells (Fig. 3D). Immunostainings confirmed that the differentiated CTO layers contained ciliated and goblet cells, and that the membrane-proximal layer primarily consisted of p63^+^ basal cells (Fig. 3E, F, G). The abovementioned TEER measurements indicated that the CTO-derived 2D cultures formed a strong barrier, and we indeed observed the expression of a selection of tight junction proteins: zonula occludens-1 (ZO-1), claudin 5, and occludin (Fig. 3F, G, H). Unfortunately, viability of CTO-derived 2D cultures was lost after two or occasionally three weeks at ALI as holes started to form in the cell layers and cells detached.

### Attachment of avian but not human influenza virus to CTO-derived epithelium

**Fig. 4:**
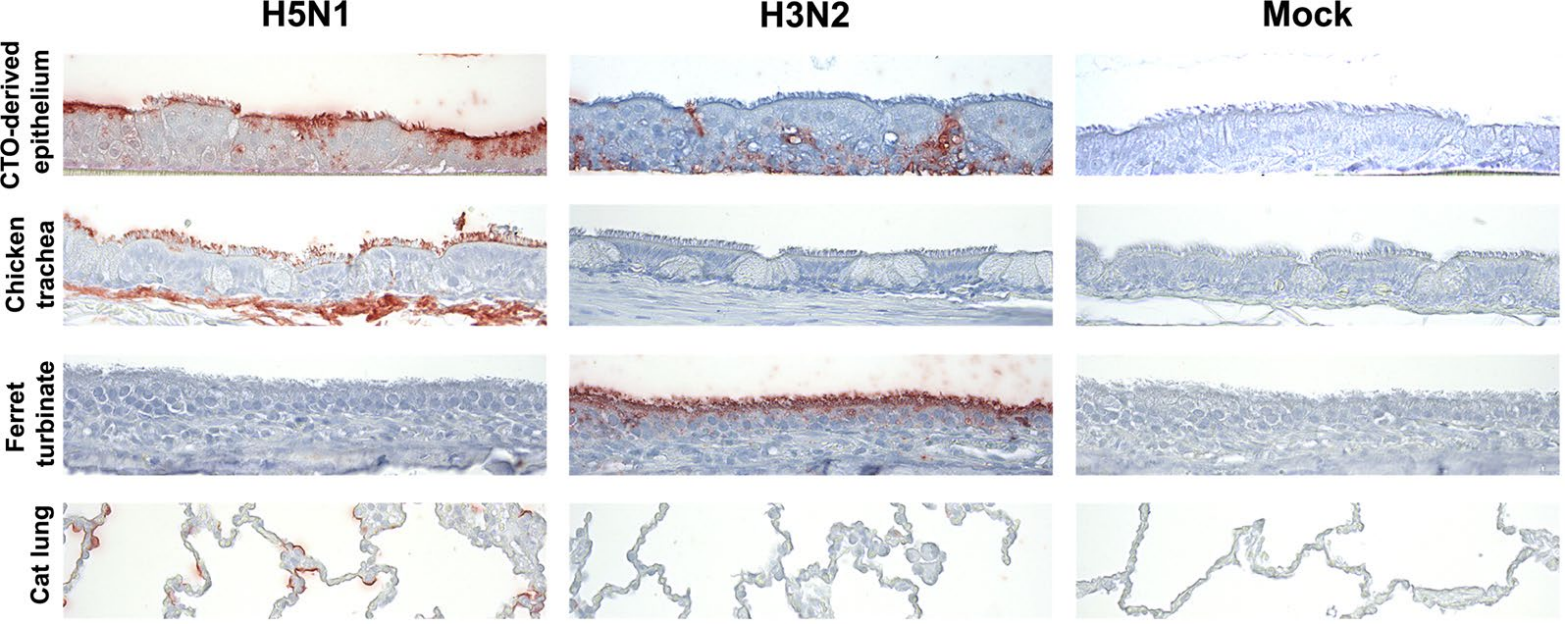
Similar attachment pattern of avian and human influenza viruses to CTO-derived 2D cultures and chicken trachea. Virus binding of H5N1 HPAIV and human H3N2 influenza virus to CTO-derived 2D cultures, adult chicken trachea, ferret nasal turbinates and cat lung was assessed by virus histochemistry. Ferret and cat respiratory tissues were included as positive control for binding of human and avian viruses, respectively. Representative images of two repetitions with red staining indicating binding of FITC-labelled virus and hematoxylin as counterstain. Pictures were taken at a magnification of 400

Influenza A viruses enter target cells by binding glycans on the cell surface that contain terminal sialic acid moieties, which have a distinct host and tissue distribution. Hemagglutinin (HA) proteins of human influenza A viruses preferably bind to α2,6-linked sialic acids, whereas AIV HAs predominantly bind to α2,3-linked sialic acids [30]. The receptor binding patterns of prototype human (seasonal H3N2) and avian (HPAIV H5N1) influenza viruses were compared between CTO-derived 2D cultures, adult chicken trachea and mammalian upper and lower respiratory tract using virus histochemistry [21]. H5N1 attached to type II pneumocytes in the domestic cat lung, but not to the ferret nasal respiratory epithelium (Fig. 4), as previously described [22, 23]. In contrast, H3N2 bound to the ferret nasal respiratory epithelium, whereas cells from the cat lung remained negative (Fig. 4), as expected [22, 23]. The CTO-derived 2D cultures showed a similar binding pattern to the chicken trachea, with H5N1 but not H3N2 binding to the apical cell layers (Fig. 4), suggesting a comparable receptor distribution between the differentiated CTOs and the adult chicken trachea.

### AIV replication in CTO-derived 2D cultures

AIVs are classified based on their pathogenicity in chickens. LPAIVs cause mild disease, accompanied by virus replication in epithelial cells of the respiratory and intestinal tracts [31]. In contrast, HPAIVs cause severe disease with high mortality rates, which is characterized by replication in endothelial cells causing hemorrhaging and edema [31]. The difference in AIV virulence is attributed to the proteolytic activation site in the viral HA protein, which is responsible for binding to and fusion with host cell membranes [32]. LPAIV HAs contain a monobasic cleavage site, which can only be cleaved by tissue-restricted proteases. HPAIV HAs contain a multibasic cleavage site (MBCS), which can be cleaved by ubiquitously expressed furin-like proteases and allows for systemic virus dissemination in chickens.

**Fig. 5:**
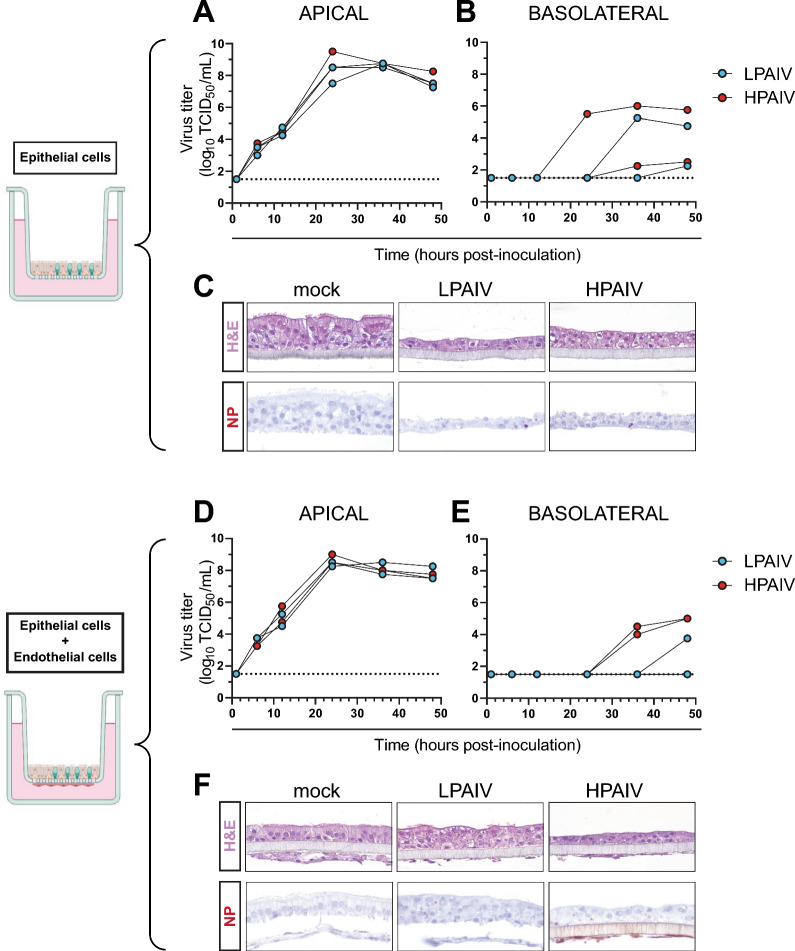
Replication of AIVs in CTO-derived 2D cultures as monoculture and co-culture with endothelial cells. CTO-derived 2D cultures (passage 7) cultured without (**A**–**C**) or with (**D**–**F**) chicken aortic endothelial cells on the basolateral side, were inoculated apically at 14 days post-seeding in duplicate with H7N7 LPAIV (HPAIV ΔMBCS) or HPAIV A/chicken/Netherlands/1/03 at an MOI of 0.0001. Detection of infectious virus titers in apical washes (**A**, **D**) and the basolateral medium (**B**, **E**) as determined by end-point titration assay in MDCK cells and expressed as log_10_ TCID_50_/mL. Each line represents a technical replicate. **C**, **F** Hematoxylin and eosin (H&E) and influenza virus nucleoprotein (NP, in dark red) stainings with hematoxylin counterstain of representative parts of the CTO-derived 2D cultures, fixed at 48 h post-inoculation and processed as formalin-fixed paraffin-embedded tissue. Created with BioRender.com

CTO-derived 2D cultures were inoculated with LPAIV and HPAIV to assess their susceptibility and ability to sustain multi-cycle replication. The epithelial cells were inoculated apically with recombinant HPAIV H7N7 A/chicken/Netherlands/1/03 [33] or recombinant LPAIV (HPAIV ΔMBCS), i.e., H7N7 A/chicken/Netherlands/1/03 from which the MBCS was reverted to the H7 LPAIV consensus cleavage site sequence, at a multiplicity of infection (MOI) of 0.0001. Infectious virus production was determined upon virus titration of apical washes, revealing comparable high levels of virus replication of both HPAIV and LPAIV (Fig. 5A). This indicated that CTO-derived 2D cultures expressed both LPAIV- and HPAIV-HA activating proteases. Concurrently, infectious virus levels in the basolateral compartment were measured, where both viruses were detected (Fig. 5B). Interestingly, histological analysis of the epithelial cell layer at 48 h post-inoculation (hpi) showed an intact cell layer (Fig. 5C). This suggested that presence of virus in the basolateral compartment was not caused by a total loss of the epithelial cell layer.

Immunostainings targeting the AIV nucleoprotein (NP) were performed on formalin-fixed paraffin-embedded CTO-derived 2D cultures at 48 hpi to assess viral antigen expression and virus tropism. Although titers were high at 1 dpi, NP-positive epithelial cells were rarely detected at 2 dpi (Fig. 5C). This might indicate that the virus-infected cells have been sloughed off, which corresponded with the visually thinner virus-infected cell layers. Furthermore, we observed a reduction in number of ciliated cells in the virus-infected versus mock conditions.

The hallmark of HPAIV disease in chickens is the preferential virus replication in endothelial cells. To mimic this phenomenon in vitro, primary chicken aortic endothelial were seeded to the basolateral side of the TWs containing CTO-derived 2D cultures and the TWs were apically inoculated with HPAIV and LPAIV. Infectious virus titers in the apical washes were comparable to those obtained upon inoculation of CTO-derived 2D monocultures (Fig. 5D). When the basolateral compartment of the co-cultures was assessed for presence of infectious virus, HPAIV was more robustly detected than LPAIV (Fig. 5E). While a few antigen-positive epithelial cells were detected in co-cultures inoculated with HPAIV or LPAIV, endothelial cells in co-cultures were NP-positive only upon inoculation with HPAIV (Fig. 5F). This suggested that the co-culture model, containing CTO-derived 2D cultures and chicken primary endothelial cells on the apical and basolateral sides of a TW respectively, is representative for the natural situation in chickens upon AIV infection, during which HPAIV replicates in the endothelium and epithelium, whereas LPAIV is restricted to replication in epithelial cell layers.

## Discussion

In this study, we describe the isolation, maintenance, and mucociliary differentiation of avian tissue-resident stem cell-derived tracheal organoids. Epithelial cells were obtained from late-stage chicken or duck embryos, which eases the process of sample acquisition as embryonated eggs are readily available, covered by less stringent ethical regulations, and less costly than adult animals. Long-term culture conditions were established for CTOs, but DTO proliferation halted at approximately six passages. We show that CTOs exhibit some degree of differentiation, but that mucociliary differentiation into pseudostratified epithelial cell layers required the culture of CTOs at ALI in Pneumacult-ALI medium for two weeks. Furthermore, we show that differentiated CTO-derived 2D cultures can be used as a model system for avian influenza virus research, as they express the adequate sialic acid receptors to allow attachment and proteases to sustain multi-cycle replication of LPAIVs and HPAIVs. HPAIV preferentially disseminated to the endothelium in epithelial/endothelial co-cultures.

The culture conditions to allow for the long-term maintenance of avian tracheal organoids were directly based on a human AO medium formulation as described by Sachs et al*.* [17]. They report, in addition to basal cells, the presence of ciliated, club, and goblet cells in AOs for at least 19 passages without the need for differentiation at ALI. The percentage of ciliated cells, whilst submerged in BME, can even be increased by maintenance in an alternative medium formulation based on Pneumacult-ALI supplemented with the Notch pathway inhibitor DAPT [18]. Here, the CTOs showed some degree of differentiation, generally containing, in addition to p63^+^ cells, muc5AC^+^ cells, but ciliated cells were not detected anymore after a couple passages. Furthermore, the CTOs converted to cystic organoids that nonetheless could undergo mucociliary differentiation upon placement at ALI up to passage 15, albeit with a reduced efficiency from passage 10 onwards. The observed long-term culture capacity of CTOs is striking as Oost et al*.* recently reported that chicken intestinal organoids were completely dependent on the addition of chicken-derived R-spondin 1, which only shows a 65% identity with the murine equivalent that we have utilized here [10]. However, snake venom gland organoids were also maintained for over six months using mammalian growth factors [34]. Yet, exchanging the mammalian factors used in the current protocols for avian-derived ones might allow an optimized preservation of stemness that is now lacking for the long-term culture of DTOs. Furthermore, orthologous human AOs cultured at ALI can be maintained for at least a couple months [35], which stands in stark contrast with the two to three weeks of viability that can be achieved with CTO-derived 2D cultures. The addition of avian growth factors, defined or undefined through co-culture with avian fibroblasts, might prolong their life span and viability. Shen et al*.* indeed observed decreased cell death and increased proliferation upon the addition of chicken embryo extract to the culture medium of avian tracheal cells [16]. Alternatively, exogenous stimulation of the Wnt signaling pathway, required for maintenance of stemness, by adding Wnt ligand or the GSK3 inhibitor CHIR99021 might benefit avian organoid culture. Wnt ligand is not a canonical supplement in AO medium because Wnt3a was reported to be produced by human AOs themselves [17]. Furthermore, exchanging the extracellular matrix (ECM) components used in the current protocols, i.e., murine BME and human fibronectin, by avian- and tissue-specific ECM might provide avian organoids with well-matched environmental cues [36].

Most cell lines on which monobasic cleavage site-containing influenza A viruses are produced in vitro require the addition of exogenous trypsin, as such cell lines do not express the adequate trypsin-like serine proteases required for HA activation. Epithelial cells of mammalian and the less researched avian respiratory and intestinal tracts readily express such trypsin-like serine proteases, allowing sustained multi-cycle replication of monobasic cleavage site-containing viruses. Here, the viral titers following inoculation at low MOI of LPAIVs and HPAIVs were comparable, indicating that the HA proteins from both variants were efficiently cleaved by trypsin-like serine proteases and ubiquitous furin-like proteases respectively. The exact nature of these HA-activating avian proteases is yet unknown, but multiple candidates have been proposed [37, 38]. Interestingly the H7N7 HPAIV was detected more abundantly in the endothelial cells following apical inoculation of an epithelial/endothelial co-culture system as compared to the LPAIV. The latter is expected as chicken primary aortic endothelial cells do not allow for productive LPAIV replication [39]. In mammalian epithelial/endothelial co-culture systems infected with human seasonal influenza viruses, viral antigen was also not detected in endothelial cells [40, 41]. In contrast to gallinaceous birds, HPAIVs are not endotheliotropic in humans and in most species of wild aquatic birds, including ducks [42]. Surprisingly, human and duck primary endothelial cells are susceptible to HPAIV in monoculture [24, 25, 38, 43]. Whether initial infection of the epithelial cell layer by HPAIVs restricts infection of the underlying endothelial cells could be investigated using these epithelial/endothelial cell co-cultures.

Collectively, avian respiratory organoids provide a promising new tool to study avian virus-host interactions in a biologically relevant, continually available, and robust model system. CTOs can be easily established from minimal amounts of primary material and, upon differentiation, contain the main cell types present in the pseudostratified epithelium of the chicken trachea. However, further research is necessary to translate the current protocols to other avian species and to other parts of the avian respiratory tract. As a proof of concept, we have shown that differentiated CTOs in monoculture or in co-culture with primary endothelial cells can be used for studying avian influenza viruses, which is warranted in the current situation of heightened global threat of HPAIVs on poultry, wildlife, and human health.

## Conclusion

Avian respiratory organoids and their 2D-differentiated equivalents are a promising new model system for studying avian host–pathogen interactions.

## Supplementary Information

Supplementary file 1.

## Data Availability

All data are provided within the manuscript and figures.
