# Supplementary material for: Long-term culture of chicken tracheal organoids for the purpose of avian influenza virus research
Source: Virol J. 2025 Apr 15;22:99. doi: 10.1186/s12985-025-02714-w (PMC11998437; doi:10.1186/s12985-025-02714-w)
Supplement: Supplementary file 1 [file 12985_2025_2714_MOESM1_ESM.pdf]

Additional File 1: step-by-step protocol for avian tracheal organoid isolation, maintenance, and differentiation at ALI

Avian tracheal organoid isolation.

Perform all procedures on ice and all centrifugation steps at 4°C. Coat plastic surfaces and tips with FCS before pipetting organoids. Thaw Cultrex Reduced Growth Factor BME Type 2 Select (R&D systems) at 4°C for a minimum of 2 hours. Centrifuge dispase before use (3 min 525xg) and use supernatant only.

1. Collect tracheas from 1-5 18-day-old chicken or 21- to 23-day-old duck embryos in ice-cold Phosphate Buffered Saline (PBS).
2. Remove connective tissue with scalpels and rinse out erythrocytes.
3. Open up trachea longitudinally.
4. Dissociate epithelial cell lining:
   - In 50 mL tube; add 100% dispase (Corning; 500 µL per embryo) and incubate pooled tracheas on ice for 30-60 min with regular tapping and shaking. Check under dissection microscope whether sheets of epithelial cells have detached. Avoid overdigestion.
5. Stop digestion reaction:
   - Add FCS to a final concentration of 10%.
   - Centrifuge pieces for 1 min at 100xg.
   - Decant supernatant.
6. Add 5 mL ice-cold basal medium with 10% FCS.
7. Further separate epithelial cells from tracheal lining by pipetting up and down using pipets in order of decreasing volume: 25 mL > 10 mL > 5 mL.
8. Remove leftover large tracheal pieces using a 100 µm sieve (Falcon).
9. Removal of single cell contaminants (fibroblasts, erythrocytes, and immune cells) by low-speed centrifugation, which will pellet the epithelial cell sheets and leave single cells in suspension:
   - Add 50 mL ice-cold PBS.
   - Centrifuge for 3 min at 100xg.
   - Decant supernatant.
10. Repeat **step 9** 5-8x until the pellet consists of almost exclusively epithelial sheets.
11. Add 10 mL basal medium and transfer cells to a 15 mL tube.
12. Centrifuge 5 min 525xg and remove supernatant completely.
13. Create BME domes while keeping all reagents on ice:
   - Based on pellet size, add BME to pellet (31 µL per dome).
   - Resuspend pellet while avoiding the formation of air bubbles.
   - Disperse 30 µL dome in well center of pre-warmed 48-well-plate.
   - Leave at room temperature (RT) for 2 min.
14. Incubate plate upside down for 15 min at 39°C.
15. Add 200 µl AO medium to the domes and PBS to surrounding wells.
16. Incubate at 39°C and 5% CO_2:
   - Refresh AO medium every 3 days.
   - Passage 1:3 each week.

Avian tracheal organoid passaging.

Perform all procedures on ice and all centrifugation steps at 4°C. Coat plastic surfaces and tips with FCS before pipetting organoids. Thaw BME at 4°C for a minimum of 2 hours. Centrifuge dispase before use (3 min 525xg) and use supernatant only.

1. Replace medium by 200 µL of 100% dispase and incubate on ice for 5 min.
2. Add ice-cold PBS and collect organoids in 15 mL tube.
3. Centrifuge 5 min 525xg.
4. Remove supernatant; organoids are still present in pelleted BME layer.
5. Resuspend organoids in 1 mL of ice-cold PBS and repeat **step 3** and **step 4** until BME has cleared.

6. Resuspend in 1 mL of ice-cold PBS and break up the organoids by repeated pipetting through FCS-coated 1 mL + 10 µL pipette tip. Pipette 10x and check under microscope.
7. Add 1-3 mL of ice-cold PBS and centrifuge slowly at 100xg for 1 min. The organoids will pellet, whereas single cell contaminants such as fibroblasts will not.
8. Remove supernatant.
   - If passage number is between 1 and 5, repeat **step 7** and **step 8** until mostly epithelial cell sheets remain in the pellet.
9. Resuspend pellet in 500 µL basal medium.
10. Follow **step 12** to **step 16** from *avian tracheal organoid isolation* protocol.

Avian tracheal organoid freezing.

Perform all procedures on ice and all centrifugation steps at 4°C. Coat plastic surfaces and tips with FCS before pipetting organoids.

1. Follow **step 1** to **step 5** of *avian tracheal organoid passaging* protocol.
2. Add 1-3 mL of ice-cold PBS and centrifuge at 100xg for 1 min. The organoids will pellet, whereas single cell contaminants such as fibroblasts will not.
3. Remove supernatant, flick pellet loose, add 300 µL/dome of Cryostor CS10 freezing medium (StemCell), and aliquot one dome per vial.
4. Cool vials to -80°C with -1°C/min.
5. Transfer vials to liquid nitrogen/-135°C for long-term storage.

Avian tracheal organoid thawing.

Perform all procedures on ice and all centrifugation steps at 4°C. Coat plastic surfaces and tips with FCS before pipetting organoids. Thaw BME at 4°C for a minimum of 2 hours.

1. Thaw vials at 37°C until an inner clump of ice remains.
2. Add organoids to 10 mL of ice-cold PBS in 15 mL tube and wash twice for 3 min at 525xg.
3. Resuspend pellet in 500 µL basal medium
4. Follow **step 12** to **step 16** from *avian tracheal organoid isolation* protocol.
   - Seed one frozen dome over 2 new domes.

Chicken tracheal organoid seeding and differentiation.

Small TWs were used during this study (Corning Costar; 24-well (6.5 mm diameter/0.4 µm pore) polycarbonate (#3413)). Centrifuge dispase before use (3 min 525xg) and use supernatant only.

<u>0 dps</u>

1. Coat 24-well TWs (0.33 cm^2) with 5 $\mu g/cm^2$ fibronectin (Merck) in PBS for minimum of 2 hours at 37°C.
2. Perform **step 1** to **step 7** of *avian tracheal organoid passaging* protocol but without coating anything with FCS, as FCS quenches TrypLE Express (ThermoFisher Scientific) activity.
3. Remove supernatant and add 1-3 mL of pre-warmed TrypLE Express.
4. Incubate in 37°C waterbath.
   - Break up organoids every 2 min with P1000 followed by P200.
   - Check under microscope whether they became single cell.
5. Repeat **step 4** until suspension contains single cells only.
6. Add FCS 1:1 to quench TrypLE Express and centrifuge 5 min 525xg.
7. Remove supernatant and resuspend pellet in 3 mL of basal medium.
8. Centrifuge 5 min 525xg to remove TrypLE completely.
9. Remove supernatant and resuspend pellet in AO medium (100 µL per harvested dome).
10. Count alive cells and seed 100,000 cells per TW in 100 µL of AO medium.
11. Add 500 µL of AO medium to the basolateral compartment and PBS to surrounding wells.
12. Incubate cells at 39°C at 5% CO_2.

<u>2 dps</u>

1. Replace medium by 1:1 PneumaCult-ALI:AO medium (200 µL apical and 500 µL basolateral).

2. Check confluency of cell layer, but usually not confluent yet.

3/4/5 dps

1. Check confluency of cell layer.
2. When confluent, place the cultures at ALI:
    - Remove medium in both compartments.
    - Add 500 µL of PneumaCult-ALI to basolateral compartment.

3/4/5-14 dps

1. Monitor cellular morphology daily and/or measure TEER.
2. Every 2 days following ALI, replace basolateral PneumaCult-ALI medium.
3. Every 6 days following ALI, wash apical compartment:
    - Add 200 µL DPBS with $Ca^{2+}$and Mg^{2+} (ThermoFisher Scientific).
    - Incubate 20 min at 39°C at 5% CO_2.
    - Remove DPBS with $Ca^{2+}$and Mg^{2+}.
